# Fecal sacs attract insects to the nest and provoke an activation of the immune system of nestlings

**DOI:** 10.1186/s12983-016-0135-3

**Published:** 2016-01-20

**Authors:** Juan Diego Ibáñez-Álamo, Francisco Ruiz-Raya, Laura Rodríguez, Manuel Soler

**Affiliations:** Behavioral and Physiological Ecology group, Centre for Ecological and Evolutionary Studies, University of Groningen, 9700 CC Groningen, The Netherlands; Department Wetland Ecology, Estación Biológica de Doñana, C.S.I.C, Avda. Américo Vespucio s/n, 41092 Sevilla, Spain; Departamento de Zoología, Facultad de Ciencias, Universidad de Granada, Avda. Fuentenueva s/n, 18071 Granada, Spain

**Keywords:** Nest sanitation, Ectoparasites, Endoparasites, H/L ratio, Common blackbird

## Abstract

**Background:**

Nest sanitation is a widespread but rarely studied behavior in birds. The most common form of nest sanitation behavior, the removal of nestling feces, has focused the discussion about which selective pressures determine this behavior. The parasitism hypothesis, which states that nestling fecal sacs attract parasites that negatively affect breeding birds, was proposed 40 years ago and is frequently cited as a demonstrated fact. But, to our knowledge, there is no previous experimental test of this hypothesis.

**Results:**

We carried out three different experiments to investigate the parasitism hypothesis. First, we used commercial McPhail traps to test for the potential attraction effect of nestling feces alone on flying insects. We found that traps with fecal sacs attracted significantly more flies (Order Diptera), but not ectoparasites, than the two control situations. Second, we used artificial blackbird (*Turdus merula*) nests to investigate the combined attraction effect of feces and nest materials on arthropods (not only flying insects). Flies, again, were the only group of arthropods significantly attracted by fecal sacs. We did not detect an effect on ectoparasites. Third, we used active blackbird nests to investigate the potential effect of nestling feces in ecto- and endoparasite loads in real nestlings. The presence of fecal sacs near blackbird nestlings did not increase the number of louse flies or chewing lice, and unexpectedly reduced the number of nests infested with mites. The endoparasite prevalence was also not affected. In contrast, feces provoked an activation of the immune system as the H/L ratio of nestlings living near excrements was significantly higher than those kept under the two control treatments.

**Conclusions:**

Surprisingly, our findings do not support the parasitism hypothesis, which suggests that parasites are not the main reason for fecal sac removal. In contrast, the attraction of flies to nestling feces, the elevation of the immune response of chicks, and the recently described antimicrobial function of the mucous covering of fecal sacs suggest that microorganisms could be responsible of this important form of parental care behavior (microbial hypothesis).

## Background

Nest sanitation, defined as the removal of any object that is not an intact and viable egg or young from the nest, is an important and widespread behavior in birds [1]. The removal of excrements, the most common form of nest sanitation, has been known for a long time (i.e. [2–4]), but still remains a neglected topic in studies of animal behavior [5, 6].

The removal of excrements has been proposed to be determined by nest predation (nest predation hypothesis; [2, 7]). However, this hypothesis has received mixed support, with studies finding that excrements increase nest predation [8] while others failed to find such relationship [9–11]. Parasitism has also been proposed to have shaped this form of parental care behavior [12] and even if it is frequently cited as a demonstrated fact (e.g. [13, 14]) or a “well known behavioral adaptation against arthropod nest parasites” [15], to our knowledge, there is no previous experimental test of this hypothesis (parasitism hypothesis). This is despite the study of parent-offspring interactions during the nesting phase that has promoted significant advances in our understanding of the evolution of parental care characteristics, some of them directed to reduce the risk of parasitism [16, 17]. Mosquitoes, ticks and other ectoparasites are known to detect vertebrates through chemical cues emanating from different avian-derived products (e.g. [18, 19]). In fact, chicken feces also seem to attract female mosquitoes (*Culex quinquefasciatus*; [20]) suggesting that nestling feces could have a similar effect. Indeed, feces removal has been considered a behavioral defense against parasites by some authors [12, 15, 21]. Another study on Blue Tits (*Cyanistes caeruleus*) suggested a relationship between ectoparasites and nest sanitation because female tits of nests parasitized by blowflies (*Protocalliphora spp.*) invested more time cleaning their nests than those of experimentally de-parasitized nests [22]. Furthermore, management of invertebrate parasites is recognized as a form of nest sanitation present in at least 15 bird species [1] which will indicate that other forms of nest sanitation (i.e. feces removal) could be also related to ectoparasites.

Vector-borne parasites induce several behavioral changes in blood-sucking insects to maximize their transmission success (e.g. [23, 24]). Thus, it is possible that nestling feces could increase the number of endoparasites in spite of their effect on ectoparasites if arthropods already infected and acting as vectors are more attracted by feces. Therefore, it seems critical to test the parasitism hypothesis to comprehend the selective pressures behind the origin and maintenance of feces removal behavior, especially given the controversy concerning the alternative nest predation hypothesis.

Here we experimentally test for the first time whether excrements produced by nestlings could attract ectoparasites, endoparasites (via infected vectors) or both to the nest. We carried out three different experiments using flying insect traps, artificial nests and natural nests of the Common Blackbird (hereafter blackbird) baited with real nestling feces and under two different control situations. The first experiment (flying insect traps) tested for the attraction of ectoparasites by nestling feces alone. According to the parasitism hypothesis, we predicted that traps with feces should attract more ectoparasites than those without feces (Prediction 1). Our second experiment (artificial nests) tested for the combined effect of nestling fecal sacs and nesting material as it is possible that there are additive effects when both elements are acting simultaneously (i.e. due to rotten nest material). This second experiment also allowed us to test our hypothesis against a broader community of arthropods (not only flying insects). We predict that artificial nests baited with feces should attract more ectoparasites than those without them (Prediction 2). Our third experiment (natural and active nests) tested for the combined effect of nestling feces, nesting material and active nestlings in the most natural situation possible, exploring the potential consequences that the presence of real blackbirds could have in the attraction of parasites. We predict that nestlings living in natural nests with feces should have higher ectoparasite loads than those of control nests (Prediction 3). Independently, nestlings from experimental natural nests should present higher endoparasite loads than those of nests without feces (Prediction 4). Finally, if the presence of feces involves an immunological cost to birds due to the elevated prevalence of endoparasite infection or ectoparasite loads, we would expect an increased immune response in chicks of nests with excrements (Prediction 5). Both ecto- and endoparasites can activate the immune system (e.g. [25, 26]).

## Results

### McPhail traps experiment

**Fig. 1:**
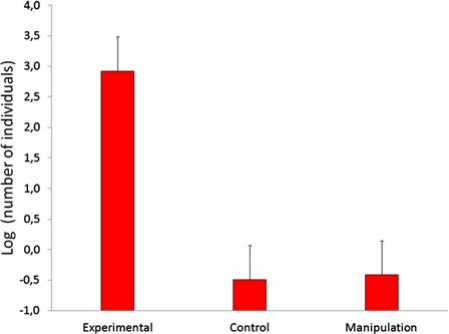
Mean number of individuals of the Order Diptera captured by McPhail traps for each treatment. *N* = 21 (seven per treatment)

We placed 21 traps in the field (seven per treatment). We captured a total of 212 arthropods of the following Orders: Diptera (92 % of captures), Arachnida (5 %), Hymenoptera (1 %), Hemiptera (1 %) and Coleoptera (1 %). Only one of these arthropods was an ectoparasite (an unidentified mosquito) captured in a manipulation control trap suggesting that nestling feces did not significantly attract ectoparasites and consequently not fitting prediction 1. That only one ectoparasites was captured prevented the use of any statistical analysis comparing ectoparasite vs non-ectoparasite prevalence. In contrast to our ectoparasite results, we found a significant effect on the attraction of Diptera (F2,17 = 12.16, *p* = 0.0005). Traps with nestling feces attracted a significantly higher number of flies than the control (Tukey HSD, *p* = 0.001) or manipulation control traps (Tukey HSD, *p* = 0.001; Fig. 1).

### Artificial nests experiment

**Fig. 2:**
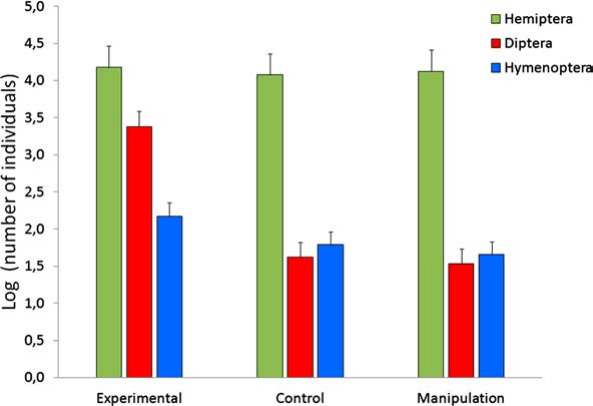
Mean number of individuals for the three main Orders of Insects captured with the artificial nest experiment per treatment. *N* = 35 (Experimental = 11; Control = 12; Manipulation control = 12)

We used 36 artificial nests for our second experiment (twelve per treatment). However, one tree with an artificial nest baited with feces was cut down in the middle of the experiment which reduced our effective sample size to 35 nests. We captured a total of 4046 arthropods distributed among the following Orders: Hemiptera (81 % of captures), Diptera (12 %), Hymenoptera (6 %) and Coleoptera (<1 %), Tisanoptera (<1 %), Dermaptera (<1 %), Lepidoptera (<1 %) and Arachnida (<1 %). None of the arthropods captured was an ectoparasite, consequently not fitting prediction 2. However, we found a significant effect for Diptera (F2,31 = 26.86, *p* < 0.000001) indicating that artificial nests baited with nestling feces attracted more flies than control (Tukey HSD, *p* = 0.0001) or manipulation control nests (Tukey HSD, *p* = 0.0001; Fig. 2). These results match with those obtained by the McPhail trap experiment. There were no significant differences for Hemiptera (F2,31 = 2.41, *p* = 0.11) or Hymenoptera (F2,31 = 0.02, *p* = 0.98).

### Natural nest experiment

A total of 63 blackbird nests were followed: 22 experimental nests, 20 control nests and 21 manipulation nests. Unfortunately, nest predation reduced our initial sample size to 33 nests that were active until the end of the nestling period (12 experimental nests, 11 control nests and 10 manipulation control nests).

We did not find significant differences for louse fly prevalence due to our experiment (χ^2^2 = 3.45, *p* = 0.18), but we found significant differences for mites prevalence among treatments (χ^2^2 = 8.29, *p* = 0.02). Blackbird nests whose attached canary nests were baited with feces were parasitized by mites less frequently (33.3 % nests with mites) than control nests (83.3 %; manual posthoc, *p* = 0.004) but not than manipulation control nests (66.7 %; manual posthoc, *p* = 0.08). There were no significant differences for feather damage produced by chewing lice among treatments (F2,20 = 1.03, *p* = 0.38). Ectoparasite results do not fit with prediction 3, moreover results about mites prevalence point out in the opposite direction.

**Fig. 3:**
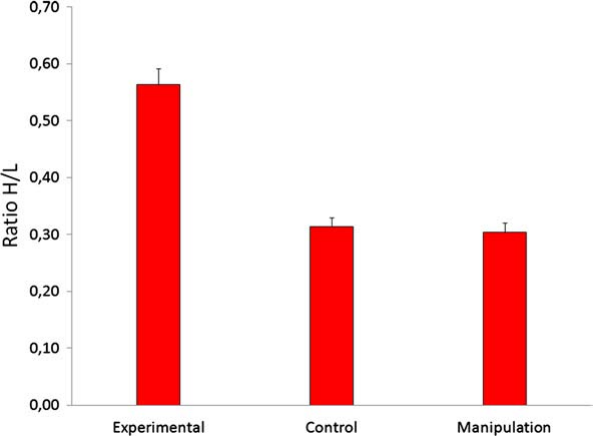
Heterophyl/lymphocyte ratio for nestlings of each treatment in the natural nest experiment. *N* = 33 (Experimental = 12; Control = 11; Manipulation control = 10)

In relation to endoparasites, we found 20 % of inspected blackbird nestlings parasitized with at least one of the targeted endoparasites. Endoparasite prevalence did not change due to our experimental manipulation (χ^2^2 = 1.43, *p* = 0.49), thus not fitting with prediction 4. In contrast, our fifth prediction was fitted as chicks living close to feces had a significant higher H/L ratio than those of control or manipulation control nests (F2,19.7 = 32.14, *p* = 0.000001; Fig. 3) indicating a higher immune response in the former. Finally, we did not find significant differences in growth rate among treatments (F2,38.8 = 1.74, *p* = 0.20), suggesting that nestling feces did not alter growth.

## Discussion

Our results do not support the parasitism hypothesis; the presence of nestling feces did not significantly attract ectoparasites in any of our three experiments testing this hypothesis in different situations. Ectoparasites were not attracted by fecal sacs alone (trap experiment), in combination with nesting material (artificial nest experiment) or, more interestingly, in the presence of active chicks (natural nest experiment). In fact, no ectoparasite was captured in experimental traps or artificial nests (those baited with feces), even if they significantly attracted other arthropods. To our knowledge, this is the first time that this hypothesis has been experimentally tested even though it was proposed long ago [12].

A previous study [20] found that female mosquitoes were attracted by the feces of adult chickens, while we found no attraction when testing this effect for nestling feces. These differences could be explained by the different structure of adult and nestling excrements. Nestlings, but not adults, produce feces encapsulated in a mucous covering that is known to isolate bacteria within these so-called fecal sacs [27]. It is also possible that this mucous covering could prevent the dispersal of chemical cues of the excrements that might attract ectoparasites. It should be interesting to test the role of this mucous covering in the context of the parasitism hypothesis in future studies. Other parental behaviors, like active removal of ectoparasites, has been suggested to play an important role in nest sanitation [1, 22, 28] and could compensate for the attraction of ectoparasites by nestling feces. However, it is unlikely that parental activity will be the reason for not finding significant differences in ectoparasite loads among treatments of our natural nest experiment given that we also did not find attraction of ectoparasites in the other two experiments which tested this hypothesis in the absence of parents.

Interestingly, we found a significant reduction in the prevalence of mites due to the presence of fecal sacs. Duffy [29, 30] proposed that seabirds could use excrements to control nest parasites, in a similar way as some bird species use secondary plant compounds (reviewed in [31]). He suggested that seabird guano, ammoniacal and cement-like, might discourage parasites which could be the reason why seabirds tend to place nests near seabird guano [29, 30]. Uric acid is a common compound in birds’ feces which is quickly transformed into CO_2_ and ammonia by specialized microorganisms once out of the body [32]. For instance, ammonia is known to repel different species of ticks [33], and might repel also other kind of Acari like mites. But, in the blackbird [9], like in other passerines [34], no fecal sac is left in the nest indicating that any potential benefit obtained from fecal sacs repelling mites will not surpass the costs of not removing them from the nest, at least in this species.

Our results on endoparasite prevalence also do not support the parasitism hypothesis because the percentage of nests with infected nestlings did not increase in the presence of fecal sacs. However, the implications of this result should be taken with caution. First, it is still possible that the cost of nest sanitation is related to parents rather than offspring. Second, we cannot discard the possibility that nestlings from our experimental natural nests were more infected than those from control or manipulation control nests during the last stage of the nestling period, and that this differential infection was still undetectable when we sampled our birds because it has not progressed far enough to be detectable in peripheral blood [35, 36]. As blackbird chicks grew older, there was more fecal material in the attached canary nest, simulating a natural situation in which there was no removal of nestling feces. There could be a threshold of fecal material volume above which vectors are attracted. However, the existence of such a threshold is unlikely given that small amounts of feces (6 g) in our McPhail traps experiment, corresponding to the quantity of feces of 3 days old blackbird chicks in our natural nest experiment, significantly attracted other insects like flies.

We found that feces increased the H/L ratio of nestlings suggesting that our experiment did have an effect in the immunology of blackbird nestlings. To our knowledge, this is the first evidence showing a relationship between fecal sac removal and immune response in nestlings. However, the cause of this change is not clear. An elevated H/L ratio has been related to challenges to the immune system (e.g. [37]), and a recent study has found a positive correlation between *Plasmodium sp*. infection and H/L ratio in blackbirds [26]. The elevated H/L ratio observed in nestlings living near their feces could indicate that they were indeed more parasitized by endoparasites (even if they have not been detected in blood smears). On the other hand, some studies have found a positive relationship between blood-sucking mites living in the nests of birds (family Dermanissydae) and H/L ratio [38, 39]. It is therefore possible that the observed change in mite loads could explain the increased H/L ratio of experimental nestlings, but this result could also reflect an increase in bacterial infection in these nestlings which have also been related to the elevation in the H/L ratio of birds (e.g. [40, 41]). In fact, our results from the trap and artificial nest experiments indicating that flies are significantly more attracted to fecal sacs support this reasoning. The feces of nestlings includes pathogenic bacteria [42, 43] and it is known that the mucous covering of fecal sacs act as a temporal protective barrier against microorganisms from the inside of nestling feces in this species [27]. Furthermore, flies can play an important role as vectors for bacteria [44, 45]. Thus, it is possible that flies attracted to fecal sacs deposited in the attached canary nest transport potentially harmful microorganisms from excrements to blackbird chicks living close by, which activated their immune system to fight against them. Nevertheless, increases in the H/L ratio have also been related to different physiologically stressing situations (e.g. [46, 47]). We cannot rule out that living near feces could affect the physiology of nestlings in an unknown way, for example if parental behavior is altered by the presence of fecal sacs (i.e. brooding behavior). Different species of passerines are known to detect foreign odors at the nest and modify their behaviors accordingly (e.g. [48–50]. Thus, the intense odor associated to nestling feces could disturb adult blackbirds, which consequently could change some aspects of their behavior (i.e. the time they spend at the nest).

It is also important to mention that our experimental manipulation did not affect growth rate in blackbird chicks, even if it produced changes in their immune response. There are two possible explanations for this result. First, the increase in immune response may be restricted to the last part of the nestling period and, thus, its influence is not long enough to affect growth rate. Second, the induced changes in immune response due to living close to fecal sacs (but not touching them) are not big enough to impair growth. Both reasons are based on the existence of a trade-off between the immune system and growth rate (e.g. [51, 52]). However, some researchers have shown that such a negative relationship only appears when food is scarce [53]. According to this last scenario and considering that food limitation is low in our studied population [54], we could explain that our manipulation modified nestlings’ immune response but not growth rate.

## Conclusions

Our results point out that parasitism does not seem to play an important role in the evolution of the removal of nestling feces contrasting with previous assumptions (e.g. [14, 15]). Nest predation seems to be of little relevance regarding nest sanitation behavior too [7, 9–11] although appropriate experiments to test for the potential nest predator attraction of the visual component of fecal sacs are lacking. On the one hand, it is possible that nest predation or parasitism could originate and fix this behavior in a common ancestor of passerines, but that these selective pressures are not acting anymore. The removal of nestling feces could have been maintained in many bird species, like blackbirds, due to its low costs (i.e. in terms of energy or time). On the other hand, there could be other selective pressures determining this important form of parental care behavior. Previous work about the importance of microorganisms in relation to avian nests (e.g. [55–57]) and in relation to nest sanitation [27] imply that the removal of nestling fecal sacs from nests could be determined by the risk of infection by harmful microorganisms (the microbial hypothesis). Some findings of this study support this new hypothesis (attraction of vectors of potentially harmful microorganisms and the elevation of H/L ratio) and highlight that more investigations on this topic and with other species are needed to fully comprehend this poorly understood but conspicuous behavior in birds.

## Methods

### Study area and species

This study was carried out during the springs of 2012 and 2013 in the Valley of Lecrín, south of Spain (36°56' N, 3° 33' W; 580 m.a.s.l.). The study area is dominated by orange groves in which blackbirds usually nest (see [54] for a more detailed description of the breeding population). We used the blackbird as the model species because (i) adults remove all excrements produced by their nestlings from their nests [11], (ii) their endoparasites are well known (e.g. [36, 58, 59]), and (iii) we already have information about the ectoparasites that affect their nestlings. For example, we have previously identified the louse fly species parasitizing blackbird nestlings in our population (*Ornithomyia avicularia*; Miguel Carles-Tolra pers. comm.) and the community of nest mites is already known, mainly composed by the genus *Trichouropoda*, specially *T. ovalis* [60]. We actively searched the area for blackbird nests in both years.

### McPhail traps experiment

In May of 2012, when blackbirds were breeding in the study area and after detecting the first ectoparasites (louse flies and mites) in blackbird chicks, we placed 21 commercial McPhail traps (Econex S.L.) in orange trees. These traps are specially designed to capture flying insects in different habitats (e.g. [61, 62]). All traps were attached to the trees using hemp strings (commonly found in the area), thus avoiding potential unwanted attractive effects of other materials (i.e. colored plastic). Traps were attached at 2 m height, which is the median height of blackbird nests in this population (range 1.5 - 4 m above the ground; J.D. Ibáñez-Álamo unpubl. data). We placed traps along a transect in the study area, with 50 m of distance between traps to avoid interference among them. Blackbird nests are usually closer than 50 m in this population (J.D. Ibáñez-Álamo unpubl. data). Each trap contained a solid insecticide tablet (2.2 dichlorovinyl dimethyl phosphate) to kill all insects trapped. This is a colorless and odorless insecticide commonly used to control insects in agriculture and pest management [63].

Our experimental design consisted in three groups: (i) Experimental traps with fresh nestling feces obtained from blackbird chicks during the previous 3 h; (ii) Control traps baited with mud (a mix of water and earth collected from the surrounding area; (iii) Manipulation control traps with just the insecticide tablet to control for the potential attraction effect of our traps for arthropods. We randomly assigned each trap to one of the three treatments avoiding repetitions of consecutive traps. We visited all traps approximately every 72 h in order to collect arthropods captured, thus, avoiding the potential attraction of scavengers to the traps, and to add new fresh material (feces or mud). Doing that, we simulated the natural accumulation of excrements along the nestling period due to the natural feces production of real chicks. We collected fresh excrements from nestlings of close active nests. The mean quantity of feces added to experimental traps for the whole period (6.0 ± 0.1 g) did not differ significantly from the mean quantity of mud added (5.9 ± 0.1 g; ANOVA F1,11 = 1.07, *p* = 0.32). Each trap was active for 10 days, a period which is very similar to the mean nestling period of blackbirds in the population (11.8 ± 0.3 days; [54]).

We quantified and identified to the level of Order all arthropods trapped (following the criteria indicated in [64]), and determined those that are known ectoparasites (i.e. mosquitoes within Diptera). McPhail traps baited with different material are widespread in insect studies, and provide comparable data on flying arthropods abundance [61, 62].

### Artificial nest experiment

In March of 2013, we collected blackbird nests used only for the incubation stage that had been predated or abandoned. We avoided collecting nests that contained nestlings to eliminate the potential effect of any substance produced by chicks that was impregnated in the nest and could have masked the attraction effect of excrements and nesting material alone. The exact location of the nest was marked with a GPS (Garmin Gekko) and a small piece of string attached to the branch. We collected nests and transported them in independent hermetic plastic bags to the lab. The nests were kept at 50 °C and in obscurity for 48 h in an oven in order to kill all arthropods that were already in the nests [65].

The nests where placed in their original locations the last week of April, when most active blackbird nests have nestlings. We placed a Petri dish (85 mm diameter) with a small hole (18 mm diameter) on top of the nest fitting exactly with the inside diameter of the nest. Just after the placement of the modified Petri dish, we extended its surface with a non-odor, transparent and environmental resistant adhesive (Temobi, Impex Europa S.L.). Our experimental design was similar to that of the McPhail insect traps and consisted in three groups: (i) Experimental treatment: nests baited with nestling feces obtained from blackbird chicks during the previous 3 h; (ii) Control treatment: nests baited with mud (a mix of water and earth collected from the surroundings of the nest); (iii) Manipulation control treatment: nests with just the Petri dish and the adhesive to control for their effects on the attraction of arthropods. We randomly assigned each nest to one of these groups. The small hole in the dish allowed the odor of the feces to disperse and avoided the proliferation of fungus which could attract other arthropods to the trap. We visited all nests every 72 h in order to change the Petri dishes to avoid the potential attraction of scavengers to the traps, and to add fresh material. With this schedule, we simulated the natural accumulation of excrements along the nestling period due to the natural feces production of real chicks. The mean quantity of feces added to experimental nests for the whole nestling period (9.7 ± 1.5 g) did not differ significantly from the mean quantity of mud added (9.6 ± 1.5 g; ANOVA F1,23 = 0.94, *p* = 0.90). The design of this artificial nest traps allowed us to collect flying arthropods (like with McPhail traps), but also non-flying arthropods, which allow us to test the potential attraction of nestling feces against a broader variety of arthropods/ectoparasites.

We stopped the experiment after a period of 12 days, which match with the mean nestling period of blackbirds in this population (11.8 ± 0.3 days; [54]). Then, we quantified and identified to the level of Order the arthropods trapped in the adhesive traps following the criteria indicated in [64], and determined those that are known to be ectoparasites (i.e. mosquitoes within Diptera).

### Natural nest experiment

During the breeding season of 2012, we attached a commercial Canary (*Serinus canaria*) nest made of vegetable fiber below each natural blackbird nest using hemp strings following the procedure described in [10]. We placed the Canary nest just after the hatching of the whole clutch.

We created three different treatments based on the material included into the attached nest: (i) Experimental nests with nestling feces; (ii) Control nests with mud simulating the same consistency as natural excrements; and (iii) Manipulation control nests with nothing added. Each nest was randomly assigned to one of the treatments to avoid potential effects of parental and territory quality on the attraction of ectoparasites.

We visited all nests every two days (5 visits per nest) during the complete nestling period in order to add new fresh material to the attached nest. Similarly to the two previous experiments, we simulated the natural production of excrements along the nestling period. There were no significant differences in the mean quantity of feces added to experimental nests (17.9 ± 1.5 g) or mud added to control nests (15.9 ± 1.5 g; ANOVA F1,24 = 0.94, *p* = 0.34). We collected body weight measurements for each nestling during our first and last visit in order to calculate growth rate based on [66]. Basically, we used the residuals of a linear regression of the last visit body weight on the first visit body weight.

During the fifth visit, when blackbird nestlings were 11 days old, we carefully inspected the chicks for ectoparasites. Once at the nest, we quickly introduced the nestlings in a white cotton bag and checked the bird for louse flies (Hippoboscidae) following the recommendations provided by [67]. After this first examination, we noted the presence/absence of mites in the bag and counted the number of feather damage (holes) produced by chewing lice on both wings [67].

We then obtained a blood smear for each nestling present in the nest using the standard two-slide wedge procedure. We air dried the smears, fixed them in absolute ethanol for 5 min and stained them with Giemsa solution for 40 min [68]. We examined blood smears looking for vector-borned parasites, specifically for extracellular parasites (*Trypanosoma sp* and *Microfilaria sp*) scanning 300 fields at 200x magnification, and intracellular parasites (*Haemoproteus sp* and *Plasmodium sp*) examining 10,000 erythrocytes per smear under the 1000x magnification and oil immersion [36, 69]. We also obtained counts of lymphocytes and granulocytes per 100 leukocytes and calculated the heterophils/lymphocytes (H/L) ratio [37]. This ratio provides useful information of the immune status of birds at the time of sampling (e.g. [37, 41, 70]. Determinations of endoparasites and H/L ratio were done without knowing the origin of the samples, which allow to avoid important bias frequently observed in experimental studies [71–73].

We decided not to quantify arthropods in this experiment because it would have involved removing them from the location, thus, preventing us from detecting any effect on nestlings’ health or parasite loads (i.e. if vectors were captured before biting a nestling and infecting him).

### Statistical analyses

We carried out General or Generalized Linear Models depending on the nature of the dependent variable in order to test the effect of our treatment. We included date as a covariate in all analyses, and brood size for the analyses of the third experiment (natural nests) because the number of nestlings could be related to parasite loads [74, 75]. To test the attraction effect of nestling feces in our McPhail traps we used number of individuals for each group of arthropods or functional groups (ectoparasites vs non-ectoparasites) collected at the end of the experiment as the dependent variable. We used the same variable in order to test for the combined effect of excrements and nesting material (artificial nest experiment). In relation to the natural nest experiment, we used louse flies and mites prevalence (proportion of infested nests; [76]) and the mean number of holes per nestling for each nest. Given to the low number of infected chicks with endoparasites, we also used endoparasite prevalence (percentage of nests with at least one of its chicks infected by one of our targeted endoparasites) as the dependent variable to investigate the effect of our treatment in endoparasite infection. To test for the effect of nestling feces on the immune response or growth rate of chicks we carried out general linear mixed models including H/L ratio or growth rate as dependent variable and nest identity as random factor. We systematically checked the assumptions underlying the use of these analyses (normality and homoscedasticity) and log_10_-transformed our dependent variables when necessary. We used Tukey HSD posthocs for comparisons among treatments in general linear models and manual posthocs (independent comparisons among pairs of treatments) in generalized linear models. All statistical analyses were performed using STATISTICA ver. 8.0 software (StatSoft Inc. Tulsa, OK, USA). The values are reported as means ± SE.

#### Ethical note

This research was conducted according to national (Real Decreto 1201/2005, de 10 de Octubre) and regional (permissions provided yearly by Consejería de Medio Ambiente de la Junta de Andalucía) guidelines.
